# A large intrathoracic extramedullary hematopoiesis in alpha-thalassemia: A case report

**DOI:** 10.1097/MD.0000000000017612

**Published:** 2019-11-01

**Authors:** Jianan Chen, Yuan-Ling Liu, Min-zhen Lu, Xing-Lin Gao

**Affiliations:** a Shantou University Medical College; Department of Respiratory and Critical Medicine, Guangdong Provincial People's Hospital, Guangzhou; b Department of Respiratory and Critical Medicine, Guangdong Geriaitric Institute, Guangdong Provincial People's Hospital, PR China.

**Keywords:** alpha-thalassemia, CT-guided percutaneous mediastinum biopsy, Extramedullary hematopoiesis, mediastinal neoplasms

## Abstract

**Rationale:**

Extramedullary hematopoiesis (EMH) is a rare disease characterized by the formation of hematopoietic elements outside the bone marrow driven by several hematological disease. To the best of our knowledge, EMH is relatively common in patient with beta-thalassemia or hereditary spherocytosis but rarely reported in patients with alpha-thalassemia. Here, we discuss a large intrathoracic EMH (measuring 95 mm × 66 mm) without presenting severe complications in alpha-thalassemia along with literature review.

**Patient concerns:**

A 55-year-old Chinese female patient with alpha-thalassemia presented with ipsilateral pleural effusion and low hemoglobin level.

**Diagnosis:**

Lung cancer was suspected at first and the mass was subjected to CT-guided percutaneous mediastinum biopsy and the pathology confirmed the final diagnosis of extramedullary hematopoiesis.

**Interventions:**

Blood transfusion, thoracentesis and regular follow up were scheduled rather than surgical interventions or radiotherapy since our patient did not exhibit significant symptoms.

**Outcomes:**

After 6 months’ regular follow up, the patient exhibited no evidence of disease progress.

**Lessons:**

EMH is frequently misdiagnosed and should be differentiated from other masses in thoracic cavity, especially when the underlying hematological disease is discovered. Treatment methods of EMH include surgical resection, hyper-transfusion, hydroxyurea, low-dose radiation or a combination of them.

## 1 Introduction

Extramedullary hematopoiesis (EMH) is a rare and benign entity that defined as the proliferation of hematopoietic tissue occurring outside the bone marrow, often secondary to several types of hematological diseases.^[1]^ The exact mechanism of this development is still unclear, most likely related to the compensatory hematopoiesis. Alpha-thalassemia is inherited as an autosomal recessive disorder characterized by a microcytic hypochromic anemia, and is a result of impairing production of alpha chains from 1, 2, 3, or all 4 of the alpha globin genes, leading to an aberrant hemoglobin structure and oxygen carrying ability. As a compensatory response in various anemias, EMH may develop with thalassemia.^[1–3]^ Herein we report a case of intrathoracic EMH in a 55-year-old Chinese female patient complicating with alpha-thalassemia, manifesting as a large mass in postero-inferior mediastinum of the paravertebral region which was initially suspected as a lung cancer. To the best of our knowledge, very few cases of extramedullary hematopoiesis in patient with alpha-thalassemia have been reported till date and such a large mass (measuring 95mm × 66 mm) has not previously been described.^[1–4]^

## 2 Case report

**Figure 1:**
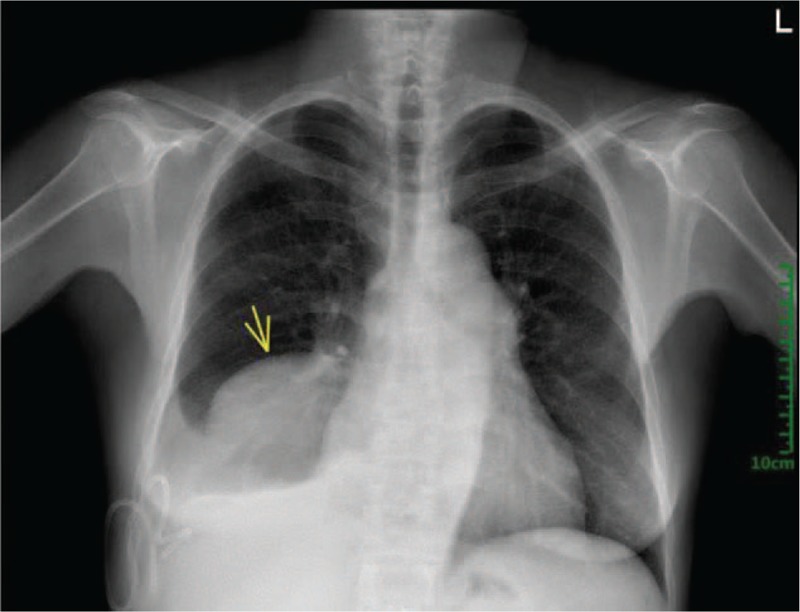
Chest X-ray findings. A large soft tissue density mass (yellow arrow) in the posterior right lower thorax on a posteroanterior chest radiography.

**Figure 2:**
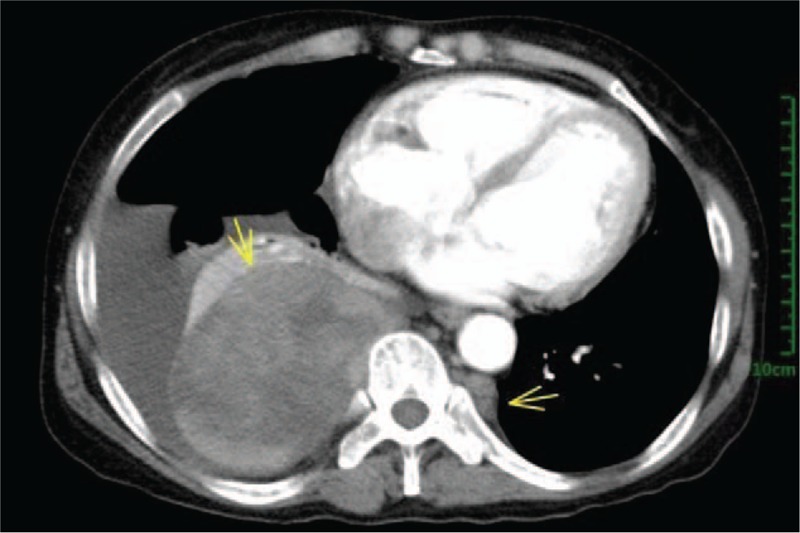
Chest CT findings. Extramedullary hematopoiesis present as a 95 mm × 66 mm well-marginated mass (white arrow) in the postero-inferior mediastinum on a chest computed tomography scan.

**Figure 3:**
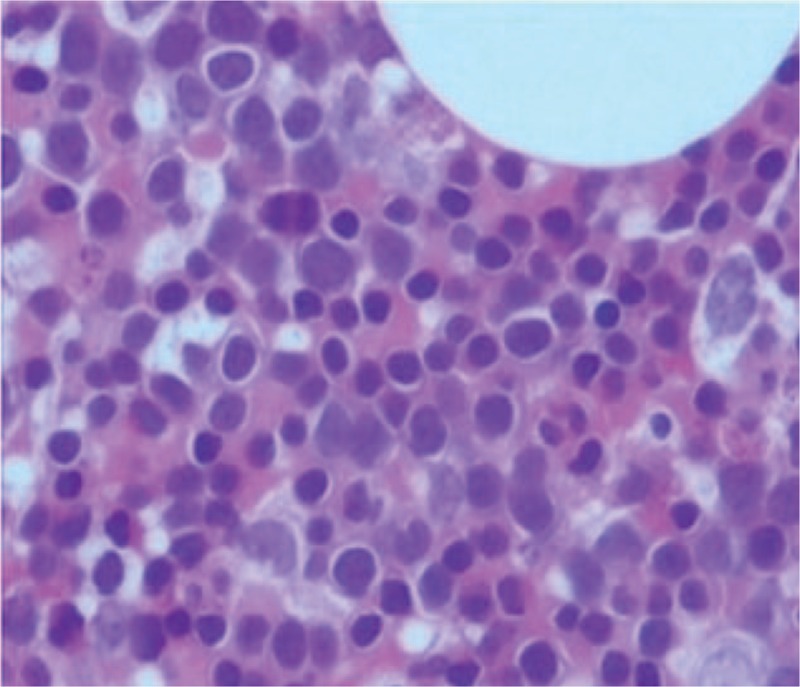
Bone marrow aspirate analysis. A bone marrow aspirate analysis revealed bone marrow especially erythroid is hyperplasia (G: E was decreased, the relative proportion of immature red blood cells was also increased), and adipose tissue accounts for 15% of the medulla area.

A 55-year-old female patient with a history of alpha-thalassemia was admitted to the department of Respiratory Medicine with a 4-month history of productive cough, shortness of breath, and fatigue. There was no other specific past medical history. The vital signs were normal; On her physical examination, there could be examined hepatosplenomegaly on palpation, but no other specific abnormalities were identified other than a pale conjunctiva and icteric sclera. The routine blood tests on admission were as followed: Hemoglobin 62 g/L(reference value: 12.0–16.0 g/L), Hematocrit 24.2% (reference value: 36%–46%), MCV 79.6 fL(reference value: 80–100 fL), Platelets 227 × 10^9^/L(reference value: 150–400 × 10^9^/L), Reticulocyte count 0.084% of red cells(reference value: 0.5% to 1.5% of red cells). Chemistry analysis findings were not specific. Despite slightly increasing Neuron-Specific Enolase (NSE) and CA-125 in our case, no further evident supporting the diagnosis of lung cancer has been found. Incidentally, a large soft tissue density oval-shaped mass was found in the right lower area of thorax on a chest radiography (Fig. 1). Then, a thorax contrast-enhanced Computed Tomography (CT) scan has been down to figure out more details about it and the result revealed a well-marginated oval soft tissue mass (Fig. 2) (largest one measuring 95mm × 66 mm at the maximum diameter, with a mild heterogeneous enhancement) in the right postero-inferior mediastinum with right pleural effusion and local atelectasis of the right lung, with no intrusion into or widening of the neural foramina and no cortical erosion of the ribs or vertebral bodies. Another mass was located at the left paravertebral region. Marked hepatosplenomegaly was also revealed in the CT. The bone marrow aspirate analysis was done and demonstrated the erythroid hyperplasia (G: E has decreased) and the relative portion of immature red blood cells (intermediate and late stage) was increased (Fig. 3).

**Figure 4:**
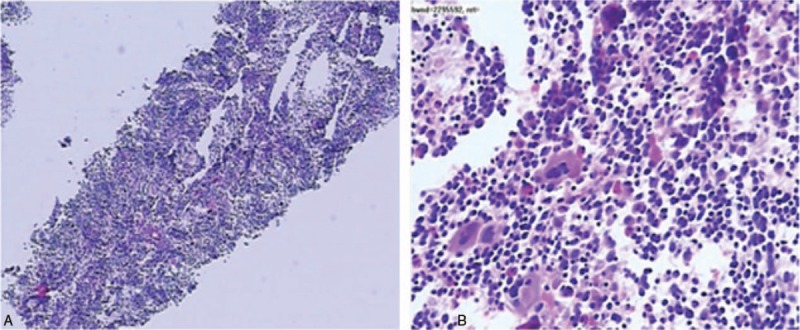
A and B. Percutaneous mediastinum biopsy. The main components were hematopoietic tissue, including hematopoietic cell lines, erythrocytes and megakaryocytes. Furthermore, G: E was decreased and the erythroid lineage was obviously proliferated, which are mainly in the intermediate and late juvenile stage, while the number and morphology of megakaryocytes were normal, and no lung tissue was found. 1. Harteveld, CL and Higgs DR, *Alpha-thalassaemia.* Orphanet J Rare Dis, 2010. 5:13. 2. Niggemann, P., et al., *Fifteen-year follow-up of a patient with beta thalassaemia and extramedullary haematopoietic tissue compressing the spinal cord.* Neuroradiology, 2005. 47(4):263–6. 3. Tai SM, et al., *Successful treatment of spinal cord compression secondary to extramedullary hematopoietic mass by hypertransfusion in a patient with thalassemia major.* Pediatr Hematol Oncol, 2006. 23(4):317–21.

Subsequently, a CT-guided percutaneous mediastinum biopsy was performed by the surgeon. Pathological biopsy analysis (Fig. 4A and B) revealed the main components were hematopoietic tissue, including hematopoietic cell lines, erythrocytes and megakaryocytes. Furthermore, G: E was decreased and the erythroid lineage was obviously proliferated, which are mainly in the intermediate and late juvenile stage, while the number and morphology of megakaryocytes were normal, and no lung tissue was found. The result was consistent with the pathologic changes of bone marrow biopsy. The pathologic biopsy and enhanced-CT findings, along with a history of alpha-thalassemia, confirmed the diagnosis of the mass representing intrathoracic extramedullary hematopoietic tumor. Blood transfusion, thoracentesis and regular follow up were scheduled rather than surgical interventions or radiotherapy since our patient did not exhibit significant symptoms. After 6 months’ regular follow up, the patient exhibited no evidence of disease progress.

## 3 Discussion

EMH refers to the formation and growth of hematopoietic elements outside the bone marrow, secondary to the insufficient erythropoiesis like myelofibrosis with myeloid metaplasia and thalassemia, also associating with many other disorders including hereditary spherocytosis, sickle cell anemia, congenital dyserythropoietic anemia, immune thrombocytopenic purpura, chronic myeloid leukemia, polycythemia vera, myelodysplastic syndrome, Paget disease, osteopetrosis, and Gaucher disease, and treatment with myeloid growth factors.^[5]^ It is a normal process in fetal life but is considered as an abnormal occurrence after birth. Common locations for EMH are the liver, spleen, lymph nodes, occasionally does EMH involve the pleural space, pre-sacral, retrosternal, sinonasal tract, temporal bone and mesentery.^[6–9]^ Intrathoracic EMH masses are generally located in the posterior mediastinum but can also manifest as interstitial pulmonary abnormality, pleural mass or haemothorax, pleural effusion, chylothorax^[10]^ and spinal cord compression either alone or in combination. EMH can be single or multiple, smooth and lobulated and may present bilaterally. For a primary mediastinal mass, it often clinically and radiologically confused with other mediastinal tumors, benign or malignant especially when the underlying hematologic disease is undiagnosed. Thymoma, lymphomas, teratoid tumors, and thyroid originated masses always stay in the anterior mediastinum.^[11]^ Chest tumor-like extramedullary hematopoietic masses are always located in the posterior mediastinum, which should be differentiated from the posterior mediastinal masses including neurogenic tumors, lymphoma, meningocele, esophageal cysts, gastric herniation, mesenchymal tumors, metastasis, and paraspinous abscess.^[12]^

In the present study, our patient only present mild respiratory symptoms, pleural effusion and anemia. Intrathoracic EMH is usually detected incidentally on chest radiographs or when it causes spinal cord compression. Magnetic resonance imaging (MRI) is one of the method of choices for diagnosing EMB especially complicating with the spinal cord compression. Most of the cases in T1 and T2 weighted MRI imaging perform iso- to hyper-intensity signals with a uniform enhancement after injecting contrast agents due to the existence of fat tissue or increased cellularity in marrow space.^[13]^ A few cases reported the 18F-PET/ CT and Technetium-99m sulfur colloid imaging (99m Tc-SC scintigraphy) used as a noninvasive modality to diagnose extramedullary hematopoiesis.^[12]^

Biopsy remains the gold standard for establishing a tissue diagnosis, and usually achieve the samples via video assisted thoracoscopic surgery (VATS)^[1]^ or CT-guided or EUS-guided fine-needle aspiration.^[14]^ Our case was diagnosed by CT-guided percutaneous mediastinum biopsy, which is an effective, highly accurate, and safe method of obtaining tissue for the diagnosis of indeterminate pulmonary lesions.

Treatments of intrathoracic EMH include regular following up, hyper-transfusion, radiation therapy, surgical excision and decompression or a combination of them. Asymptomatic disease may require no specific treatment, whereas relative low dose radiation therapy is suggested in symptomatic cases because the hematopoietic tissue is notably radiosensitive and can lead to marked shrinkage of the mass and rapid neurologic improvement.^[15]^ Sufficient transfusion in EMH patient can decrease the ineffective hematopoiesis and prevent formation of EMH mass. Surgery is a treatment strategy to relieve spinal cord compression and prevent permanent neurological deficits. The disadvantages of surgical resection include high risk of bleeding, recurrence and inadequate maintenance of hemoglobin level. Another approach for treating this condition is the use of hydroxyurea (HU), but the experience is limited. HU monotherapy has proved effective in treating four cases of EMH patients with beta-thalassemia.^[16]^ As another feasible method, the combination of radiotherapy and transfusion has also been reported, either for cases of recurrence after using treatment method alone or as an initial treatment option.^[17]^

## Acknowledgments

We acknowledge the support by Guangdong Provincial People's Hospital and Shantou University Medical College.

## Author contributions

**Conceptualization:** Jianan Chen, Yuan-Ling Liu, Min-zhen Lu.

**Data curation:** Jianan Chen, Yuan-Ling Liu, Ming-zhen Lu.

**Formal analysis:** Jianan Chen, Ming-zhen Lu.

**Funding acquisition:** Jianan Chen.

**Investigation:** Jianan Chen.

**Methodology:** Jianan Chen.

**Project administration:** Jianan Chen.

**Writing – original draft:** Jianan Chen, Xing-Lin Gao.

**Writing – review & editing:** Xing-Lin Gao.
